# Control of companion animal parasites and impact on One Health

**DOI:** 10.1016/j.onehlt.2024.100679

**Published:** 2024-01-12

**Authors:** Alessio Giannelli, Manuela Schnyder, Ian Wright, Johannes Charlier

**Affiliations:** a Kreavet, Hendrik Mertensstraat 17, 9150 Kruibeke, Belgium; b Institute of Parasitology, Vetsuisse Faculty, University of Zurich, Winterthurerstrasse 266a, Zurich 8057, Switzerland; c ESCCAP UK & Ireland, PO Box 358, Malvern, Worcestershire WR14 9HQ, United Kingdom; d Mount Veterinary Practice, 1 Harris Street, Fleetwood FY7 6QX, United Kingdom

**Keywords:** Companion animals, Parasites, One health, Research gaps, Control measures

## Abstract

The last decades have witnessed an increase in the global population and movements of companion animals, contributing to changes in density and distribution of pet parasites. Control of companion animal parasites (CAPs) becomes increasingly relevant because of the intensifying human-animal bond. Parasites impact on the health of humans and their pets, but also of wildlife and the environment. We conducted a qualitative review on the current advancements, gaps and priorities for the monitoring and treatment of CAPs with a focus on securing public health. There is a need to raise awareness, coordinate global surveillance schemes and better quantify the impact of companion animal parasites on One Health.

## Highlights

• Dogs, cats and their parasites have a poorly quantified burden on One Health.
• Parasite-borne illnesses in humans may be transmitted by companion animals. Control measures should involve companion animals to increase their effectiveness.
• Use of parasiticides remains today's cornerstone to mitigate and control the animal and human health threats posed by companion animal parasites.

## 1 Companion animal parasites: a One Health issue

The last few decades have witnessed an increase in the global population of dogs and cats. This trend has notably increased during the COVID-crisis, with people valuing more and more pet companionship, as people searched for animal closeness in times of reduced social contacts [1]. In 2022, more than half of the global population housed a pet at home, and the budget spent on animal health care followed parallel increases [2]. Animal health companies thus have expanded their investments in research and development of companion animal (CA) products, enhancing innovation in the development of novel vaccines, parasiticides, diagnostics and digital technologies [3]. Parasiticides represent the second largest segment of the global animal health market, after biologicals, and account for € 7 billion in sales (23% of the market share) [4]. In this regard, the sales of medicines for pets represent 47% of the total share [5]. The market of dog and cat parasiticides has been growing over the last decade from 31% to 34% of the product portfolio and is expected to grow further by 6% from 2022 to 2027 [5]. Undeniably, pets play an important role in companionship, entertainment, and emotional support to their owners [6]. Indeed, dog or cat ownership has been linked to reduction in cardiovascular disease risk, shorter hospital stays and positive health and welfare effects in patients affected by cancers or autism [6]. However, the closer human-animal bond also leads to more frequent violation of hygiene principles such as keeping pets in the bed(room), animals licking face and wounds as well as bite and scratch accidents. This, in turn, leads to an increased risk of exposure to parasitic diseases [7], whose importance varies depending on the context in which there are evaluated.

Never before as in the last decades, the subtle connection between animal, human and environmental health has reached the attention of the public, with the concept of “One Medicine” imprinting the direction of future health policies [6]. According to this approach, the health of people, either physical or mental, is closely connected to the health of animals and the shared environment. Although the main themes of discussion influencing the global politics of decision-making bodies tend to linger on the matters of antimicrobial resistance, food safety, or environmental health, the prevention of zoonotic diseases is also a source of major concern [7]. Within this theme, CA and their parasites have an undeniable, yet poorly quantified, burden on One Health.

In this review we analyse different drivers for increased transmission risk of companion animal parasites (CAPs) to humans and contextualise them in the frame of One Health approaches, which may vary depending on the priority given to specific diseases in different geographical settings. Via exemplary parasitic diseases in CA, we aim to illustrate the challenges when imprinting the direction of future health policies for CAP control.

## 2 Relevance for pet health

**Fig. 1:**
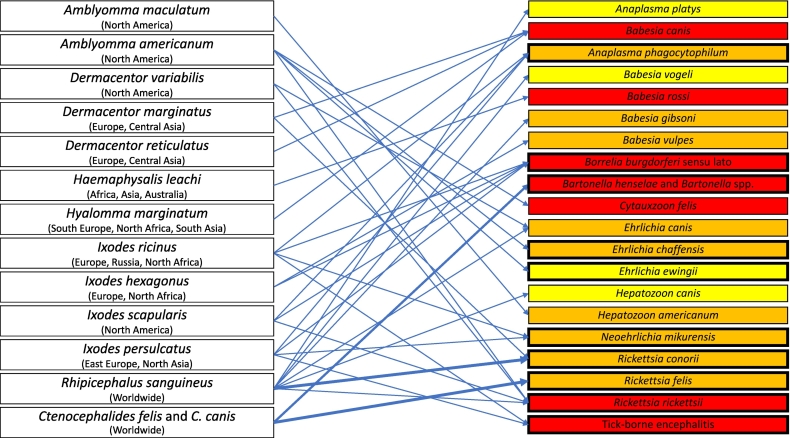
Main tick and flea species infesting dogs and cats and their vectored pathogens. Pathogen colours refer to the degree of pathogenicity in pets (yellow = mild, orange = moderate, red = high); marked boxes indicate pathogens that can also infect humans and deep blue arrows indicate the confirmed role of dogs and cats as reservoir for human infection. (For interpretation of the references to colour in this figure legend, the reader is referred to the web version of this article.)

The spectrum of *endo*- and ectoparasites affecting dogs and cats is large [8,9]. Protozoa and helminths are a significant cause of diarrhoea, respiratory issues, vomiting or weight loss in CAs (Table 1). Similarly, ectoparasites (arthropods) are a common problem to dogs and cats, causing general discomfort, anaemia, and allergic reactions or skin lesions, and potentially transmitting viruses, bacteria, protozoa, and helminths. Ticks, fleas, mosquitoes, and sand flies are all involved in the spread of the so called “vector borne diseases” (VBDs) (Fig. 1) [10,11].

**Table 1:** Main *endo*- and ectoparasites of dogs and cats, along with data on their role as reservoir for humans, geographical distribution and relevance for public health. Classification into minor or major zoonotic diseases is based on the inclusion of the parasite species in WHO control programs or its classification as a Neglected Tropical Disease.

Parasite species infecting dogs (D) and/or cats (C)	Degree of pathogenicity in D/Cδ	Zoonotic relevance for humansγ	VBDα?	Usual mode of transmission	Geographical distribution	Reference
Protozoa						
*Leishmania infantum* (D, C)	High	High (a) (NTDβ, in WHO roadmap)	Yes	Bite of infected phlebotomine sand flies, bites, wounds, blood transfusions, venereal or prenatal transmission	Mediterranean, Middle East, Central Asia, Latin America	[12]
*Trypanosoma cruzi* (D, C)	High	High (a) (NTD, in WHO roadmap)	Yes	Contact via small skin lesions or mucous membranes, ingestion and crushing infected bug vectors or eating an infected host	South America	[79]
*Giardia duodenalis* (D, C)	Moderate	Low	No	Ingestion of the cysts in undercooked, contaminated meat, accidental ingestion of oocysts in cat faeces, congenital, rarely via organ donation or blood transfusion	Worldwide	[80]
*Toxoplasma gondii* (C)	Moderate	Moderate	No	Direct contact with meat and viscera of infected animals; through food with parasitised meat and viscera or food contaminated with oocysts	Worldwide	[14]
*Cystoisospora felis, C. rivolta* (C) *C. canis, C. ohioensis, C. burrowsi* (D)	Low	No	No	Direct ingestion of sporulated oocysts or ingestion of paratenic hosts or undercooked meat	Worldwide	[81]
*Neospora caninum* (D)	Low (b)	No	No	Prenatal infection of puppies, ingestion of tissues cysts	Worldwide	[82]

Helminths						
*Opisthorchis felineus* (D, C)	High	High (a) (NTD, in WHO roadmap)	No	Ingestion of raw or undercooked fish	East Europe, Russia, Asia	[83]
*Paragonimus westermani* (D, C)	Moderate	High (a) (NTD, in WHO roadmap)	No	Ingestion of raw or undercooked crab or crayfish	Asia	[84]
*Paragonimus kellicotti* (D, C)	Moderate	High (a) (NTD, in WHO roadmap)	No	Ingestion of raw or undercooked crab or crayfish	North America	[84]
*Echinococcus. granulosus* sensu lato (D)	Low	High (NTD, in WHOroadmap)	No	Accidental ingestion of eggs	Worldwide, except Northern Europe and North America	[31]
*Echinococcus multilocularis* (D)	Low	High (NTD, in WHO roadmap)	No	Accidental ingestion of eggs	Northern hemisphere	[33]
*Ancylostoma ceylanicum,*(D, C)	Moderate	High (NTD, in WHO roadmap)	No	Ingestion of infective stages, percutaneous infection is possible	South East Asia, South Africa, Australia	[18]
*Dirofilaria immitis* (D, C)	High	Low	Yes	Bite of infected mosquitoes	Worldwide, except Scandinavia, Central and Northeastern Europe	[19]
*Dirofilaria repens* (D, C)	Low	Moderate	Yes	Bite of infected mosquitoes	Europe, Asia, Africa	[85]
*Onchocerca lupi* (D, C)	Moderate	Moderate	Unknown	Probably via a dipteran vector	Europe, United States, Middle East	[86]
*Thelazia callipaeda* (D, C)	Low	Low	Yes	Transmission via fruit flies	Europe, Far East	[87]
*Toxocara cati* (C), *T. canis* (D)	Low	Moderate	No	Ingestion of infective stages, somatic migration (prenatal) in pregnant bitches, and lactogenic transmission	Worldwide	[15]
*Ancylostoma caninum, Uncinaria. stenocephala* (D), *Ancylostoma tubaeforme* (C)	Low (c)	Moderate	No	Ingestion of infective stages, percutaneous infection is possible. *A. caninum* is also transmitted via lactogenic route	Worldwide, mainly warmer climates	[18]
*Dipylidium caninum* (D, C)	Low	Low	Yes	Ingestion of fleas	Worldwide	[88]
*Aelurostrongylus abstrusus* (C)	Moderate	No	No	Ingestion of infective stages, snails or small animals during predation	Worldwide	[89]
*Angiostrongylus vasorum* (D)	High	No	No	Ingestion of infective larvae via gastropods	Europe, certain areas of Africa and America.	[90]
*Taenia hydatigena, T. pisiformis, T. ovis, T. multiceps* (D) *T. taeniaeformis* (C)	Low	No	No	Ingestion of raw organs	Worldwide	[91]

Ectoparasites						
Ticks See Fig. 1	Variable	See Fig. 1	–	Environment	See Fig. 1	[[92], [93], [94], [95], [96], [97]]
Fleas *Ctenocephalides felis* and *C. canis* *Pulex irritans*	Variable	See Fig. 1	–	Environment	Worldwide	[13]
Lice *Trichodectes, Linognathus, Felicola*	Low to moderate	No	–	Close contact with infested animals, contaminated environment	Worldwide	[98]
Mites *Demodex, Notoedres, Otodectes, Sarcoptes, Cheyletiella, Neotrombicula*	Low to High	Low	–	Close contact with infested animals	Worldwide	[99]

α Pathogen transmitted by an arthropod vector.

β Neglected Tropical Disease; (a) In the presence of the vector, otherwise in non-endemic countries the zoonotic relevance to humans is very low.

γ Based on the severity of the symptoms, risk for contracting the infection, or a combination of these elements.

δ (b) can be high in puppies and/or in immune suppressed animals; (c) can be high in puppies.

Of all CAPs that affect humans, some can be considered of higher importance than other. However, their impact depends on the context of the assessment and can vary according to geographical distribution of the parasite, its abundance, its pathogenicity in animals or humans as well as the transmission mode between pets and humans (Table 1). There are different ways in which CAPs impact on human health, depending if the main reservoir for human infections is *(i)* the CA population (e.g., *Leishmania infantum* or fleas) [12,13]; (*ii)* the environment which was contaminated by CAs (e.g., *Toxoplasma gondii* or *Toxocara* spp.) [14,15]; *(iii)* wildlife (such as in the case of *Echinococcus multilocularis* or *Borrelia burgdorferi* sensu lato) [16,17]); or *(iv)* when there is lack of a clear parasite reservoir with pathogens that thrive equally in animals and in human hosts, with life cycles that can be maintained by transmission between humans only, such as for *Ancylostoma ceylanicum* [18]**.** The impact can also largely differ depending on whether animal or human health is considered first and foremost. For example, heartworm disease caused by *Dirofilaria immitis* is a severe disease in dogs with a limited public health importance [19]*.* In contrast, *Echinococcus granulosus* causes no disease in dogs but is a significant burden on global public health [20].

## 3 Relevance for wildlife health

Pathogens of human and domestic animal origin may infect wildlife, or vice versa, potentially resulting in outbreaks of diseases [21]. A key example includes the transmission of *E. multilocularis* from the wildlife reservoir (foxes) to dogs through common habitats with infected intermediate hosts. Dogs, in turn, represent a potential risk for alveolar echinococcosis in humans, especially in (*peri*) urban recreational environments [[22], [100]]. The progressive reduction of green areas and forests and the expansion of suburban areas brings wild animals, humans, and domestic animals in close contact with each other, with increasing risks for transmission of parasites, as in the case of Lyme disease [23]. This phenomenon was considered as a rare event in the past but is likely to become more frequent in the future [22,23]. Encroachment of human settlements and conversion of forests into rural areas increases the likelihood of encounters with ticks, known as the “crossroads effect” [23].

In addition, some CAPs may directly or indirectly affect the fitness of wildlife, potentially causing devastating impacts on biodiversity and species conservation [21]. *Toxoplasma gondii* may be a consistent danger for wildlife species which have not co-evolved with the parasite. Outbreaks of hyperacute toxoplasmosis have been reported worldwide in zoo animals and wildlife, most likely among kangaroos, wallabies, squirrel monkeys and colonies of captive lemurs [24]. Similarly, *T. gondii* causes mortality in several species of marine mammals, including threatened Southern sea otters and endangered Hawaiian monk seals [25]. The future of many of threatened species increasingly hinges on our ability to control toxoplasmosis in feral cats.

## 4 Relevance for human health

Current evaluations indicate that approximately six out of every ten known infectious diseases in humans can be spread by animals and, out of all new human pathogens detected in the last three decades, three pathogens out of four were originating from animals [26]. Neglected tropical diseases (NTDs), as defined by the World Health Organization (WHO), include a list of parasitic, bacterial and viral diseases that cause substantial illness for more than one billion people globally. At least eight of the 14 parasitic NTDs identified by the WHO (Chagas disease, leishmaniasis, echinococcosis, zoonotic hookworms, food-borne trematodes, human African trypanosomiasis, zoonotic scabies and other ectoparasites) may involve dogs or cats as potential reservoir for human infection (Table 1). Therefore, the control programmes should also involve epidemiological surveillance in CAs to increase their effectiveness. Several parasitic infections of pets have a collateral impact on human health, due to the direct or indirect transmission of the infection to humans (and vice versa) [27]. The geographical areas and socio-economic conditions where these parasitic diseases thrive differ considerably and require appropriate control measures in dogs and cats are essential to reduce the impact in humans. We shortly outline the situation of three emblematic CAPs in the following sections.

### 4.1 Zoonotic visceral leishmaniasis: lessons from Brazil

Leishmaniasis (or leishmaniosis in the context of infection in dogs) is a complex mammalian diseases caused by protozoa of the genus *Leishmania*, whose transmission to humans relies on the availability of reservoir animals and phlebotomine sand fly vectors [27]. The incidence of canine leishmaniosis (CL) varies according to the endemicity of the area, ranging from 5% to 30%, and as high as 70% in some spots [28]. Human Visceral leishmaniasis is a life-threatening disease that affects ≈200,000–400,000 persons annually and causes an estimated ≈20,000–40,000 deaths per year [29]. Zoonotic visceral leishmaniasis (ZVL) caused by *Leishmania infantum* is an important disease of humans and dogs in an area that stretches from the Mediterranean basin and Middle East to northern China, and across Central and Latin America, including Brazil [28,29]. Distribution of human cases is correlated with the occurrence of seropositive animals (dogs and other potential reservoirs) [30], which suggests that prevention of leishmaniosis in dogs has an impact on the reduction of transmission risk to humans. However, control based on culling infected dogs only has been demonstrated to be ineffective in reducing the risk for visceral leishmaniasis [[29], [30]]. This calls for integrated control methods that both (i) prevent the feeding activity of sand flies on infected hosts (e.g., through insecticide impregnated collars), and (ii) reduce parasite loads in the main reservoir host (e.g., through treatment) to scale down the incidence of ZVL. However, besides the need for vaccines with higher efficacy, the role out of effective control programmes is often hindered by socio-economic conditions and innovative control campaigns are needed to reduce *Leishmania* transmission to animals and humans.

### 4.2 Cystic echinococcosis: a remaining global problem

The genus *Echinococcus* includes several species and genotypes of zoonotic tapeworms [31]. The adult stages mostly occur in the intestine of dogs, and occasionally in cats, for both without clinical relevance. The larval stages develop in tissues of various organs of a variety of mammalian intermediate hosts, including man, for which alveolar (*E. multilocularis*) and cystic echinococcosis (*E. granulosus*) can be lethal. *Echinococcus granulosus* causing cystic echinococcosis is principally maintained within a dog-sheep cycle in pastoral regions [22]. Cystic echinococcosis is globally distributed and found in every continent except Antarctica. In endemic regions, human incidence rates can reach more than 50 per 100,000 person-years, and prevalence levels of 5%–10% may occur in parts of Argentina, Peru, East Africa, Central Asia and China [22]. The global burden of cystic echinococcosis has been estimated at 19,300 deaths and 184,000 disability-adjusted life-years (DALYs) each year, with potential underestimation of the global impact of the disease [32]. Integrated control programmes based on deworming of dogs and vaccination of lambs are known to be efficacious and can eliminate the disease in transmission zones [22,32,33], but they depend on the availability of effective implementation tools, which include control of stray dogs, slaughter supervision, public education campaigns, and routine anthelmintic treatment of dogs.

### 4.3 Fleas, households and pets

Among ectoparasites, fleas are accounted as the most frequent external parasites of CAs worldwide [34]. Due to a low degree of host-specificity, fleas of dogs and cats may have significant wildlife reservoirs. These ectoparasites have also adapted well to living in artificially heated homes being today perceived as a year-round pest [14]. The cat flea *Ctenocephalides felis* is the most abundant ectoparasite of CAs and represents the great majority of fleas in human homes. Fleas may transmit pathogens causing hemoplasmosis, rickettsiosis, dypilidiosis and bartonellosis (causing cat-scratch disease in children and immunocompromised people) [35]. However, insidious attacks by fleas on people and domestic animals causing irritation, blood loss and severe discomfort are equally important, but remain unquantified [35]. The control of a flea infestation requires a comprehensive approach on both the hosts and the environment, considering the parasites spotted on the surface of the animals represent the smallest part of the overall population, whereas the bulk of the population has typically spread to the surrounding household [14]. This means treating a flea infestation requires eliminating the parasite both on the animal and in the surrounding house.

## 5 Changing environment affecting CAP control

### 5.1 Climate change shifting parasite boundaries

Most parasites require either a suitable environment and/or intermediate hosts to complete their life cycle [36]. This makes them sensitive to environmental modifications and their distribution patterns susceptible to change [[37], [38], [39]]. Data collected over the last decade demonstrates that the epidemiology of parasitic diseases is subject to changes and will undoubtedly continue to do so in the future, with both expansions and reductions of the distribution range [40,41]. Frequently, increases in temperature enable both the parasite and their vectors to multiply faster, eventually leading to growth of the parasite infected vector population and higher disease transmission risks to humans and CAs [42].

The European Centre for Diseases Prevention and Control (ECDC), for instance, reported in 2021 new areas of presence for the mosquito species *Aedes albopictus, A. japonicus* and *A. koreicus* [43] which act as (new) competent vectors for a range of VBDs, including the canine heartworm *D. immitis*. Already in 2005, and using a climate model, a study predicted that *D. immitis* would spread into previously unaffected areas in Europe [44]. Although the prevalence has been declining throughout the world from 10.8% in 1965–1998 to 7.6% after 2016 [45], *D. immitis* is currently spreading with autochthonous cases in Central, Northeastern Europe and Siberia [46].

On the other hand, predictive models using future climate scenarios also indicate that parasite species with a limited capacity to adapt to novel environmental conditions will most likely show reduction in their distribution range. *Amblyomma* ticks, for instance, are expected to have a range reduction in Brazil due to climate warming and limitation of suitable conditions for their survival [47]. Complementary, a net decrease of areas suitable for malaria vectors has been anticipated in Africa [48]. In both cases, such contraction and expansion scenarios should not be strictly interpreted as a reduction of VBDs transmission risk but as a shifting of parasite boundaries, either at regional or global level.

### 5.2 Increased pet travel

A strengthening of the human-animal bond materialises into an increase in pets joining their owners during holidays abroad [49]. Indeed, with a valid passport, microchipping and rabies vaccination, the application of the Pet Travel Scheme (PETS) allows dogs, cats and ferrets to travel within the27 EU countries, Norway, Switzerland, or other non-European territories of the EU [49]. While on the one hand this legislative flexibility is simplifying the mobility of animals between countries, it may favour the introduction of parasites into previously non-endemic areas [50,56]. Indeed, with a few exceptions, such as the required treatment of dogs against *E. multilocularis* before entry into certified free countries*,* other parasites like *Leishmania* or ticks are prone to introduction in new areas and are not regulated or routinely monitored [50,51]. Infection with tick borne pathogens has also increased substantially in recent decades due in part to the rise in dog and cat travel [51]. In the UK, in 2005–2016, *R. sanguineus* ticks were the most common species found on animals with a history of travel mainly to Southern Europe, the USA, and the United Arab Emirates [51]. The importation of ticks to non-endemic areas due to pet travel has considerable importance for public health, as exemplified by *R. sanguineus*, atick vector and/or potential reservoir of numerous zoonotic pathogens, including *Rickettsia* species (*Rickettsia conorii* complex) (Fig. 1) [51]. The enactment of infectious diseases in previously non-endemic areas relies on the complex interaction between abiotic and biotic factors. Accordingly, pet owners need to be informed about the epidemiological situation in the area to be visited and apply adequate preventive treatments based on a risk-benefit assessment principle. Furthermore, continuous surveillance on CAs returning from abroad home should be implemented, including the search for infection with unusual parasites.

### 5.3 Changing societies

The importance of globalisation through its many facets cannot be denied in the modern era. Today, more than half of the world population lives in urban areas and this trend is expected to continue, with the urban population predicted to double its current size by 2050 [52]. Congested and overcrowded urban areas may provide a conducive environment for the dissemination of gastrointestinal parasites and ectoparasites, whereas deforestation followed by land use change alter the development opportunities of arthropod vectors [53]. Infectious disease transmission is also a function of underlying vulnerabilities of modern society. Unsustainable land use, poverty, and political instability may have measurable consequences on the dynamics of certain parasitic diseases [54]. In veterinary medicine, as an example, the relocation of sheltered dogs and cats following adoptions mediated by animal charities has a direct impact on the distribution of pathogens that may find suitable conditions enabling their survival. Data from North America provide some key examples on this phenomenon. The prevalence of canine dirofilariosis in Colorado rose from 0.5% in 2013 to 0.84% in 2017, an increase of 67.5% probably following the introduction of dogs adopted from states with a higher heartworm prevalence [55]. In Europe, 38% of dogs imported or travelling back from Southern (Spain, Italy, Greece, Turkey, France, Malta, Portugal) to Central (Germany, Switzerland, Austria) Europe were serologically *Leishmania* positive [56]. Similarly, the relocation and homing of unowned cats, while reducing animal suffering and social problems, may be linked to a higher risk of zoonotic pathogens transmission, such as *Bartonella* spp. or *Rickettsia* spp. [57]. Therefore, higher surveillance, stricter legislation as well as informed awareness of veterinarians, animal welfare organisations and animal owners importing pets from abroad along with the periodic administration of effective parasiticide treatments and adherence to basic hygiene principles are key to prevent parasites to establishing in previously unaffected areas.

## 6 Pet parasite control

### 6.1 Parasiticides

The use of parasiticides is today's cornerstone to mitigate and control the animal and human health threats posed by CAPs. Current endoparasiticides comprise benzimidazoles (e.g., fenbendazole), imidazothiazoles (i.e., levamisole), octadepsipeptides (e.g., emodepside), tetrahydropyrimidines (e.g., pyrantel) and pyrazinoisoquinolines (e.g., praziquantel). Ectoparasiticides include pyrethrins and synthetic pyrethroids, organophosphates, carbamates, formamidines, pyrazoles, neonicotinoids, spinosyns, semicarbazones, isoxazolines, and insect growth regulators. Endectocides include the macrocyclic lactones ivermectin, moxidectin, eprinomectin and milbemycin oxime [4]. The release of new parasiticides is driven by customer demands and aims to introduce formulations providing long-lasting activity, acting against both ecto- and endoparasites and with a user friendly administration route. Recent studies have also shown that anthelmintic resistance in pets should not be ignored and needs to be monitored [58,59].

Antiparasitic resistance is the genetic ability of parasites to survive treatment with an antiparasitic drug that was generally effective against those parasites in the past [59]. After an animal is treated with an antiparasitic active ingredient, the susceptible parasites are eliminated, and the resistant individuals survive to pass on resistance genes to their offspring. Increasing levels of drug resistance are documented for some human parasites, like *Leishmania* and soil-transmitted nematodes, as well as for livestock parasites like helminths [60] and *Trypanosoma*. There is no known important transfer of resistance genes from animal to human parasites or vice-versa.

In CAs, antiparasitic resistance is a known problem for heartworm *D. immitis* prevention in dogs, with macrocyclic lactone preventives showing progressively reduced efficacies since 2005 [61]. For other parasite species of dogs and cats, resistance is uncommon. However, recently the spread of multiple anthelmintic resistance in *Ancylostoma caninum* and single cases of *Dipylidium caninum* resistance against praziquantel have been shown in the US [59,62,63]. The current understanding of resistance in CAPs must be followed up with the aim to limit further onset and spread. Moreover, lessons can be learned from anthelmintic resistance research in livestock and CAs for the control of soil-transmitted helminths in humans according a One Health approach [63].

### 6.2 Diagnostics

Diagnostics provide the foundation for parasite surveillance, helping track prevalence and parasite displacement across regions, while also enabling to detect infections or evaluate the efficacy of treatments in individual animals. Although microscopy remains the cornerstone of parasitological diagnostics [64], increased availability of point-of-care tests and molecular assays in the modern era allow for more rapid and accurate diagnosis and increased sensitivity in the identification of parasitic infections. Assays detecting antigens, antibodies or DNA of parasites are now routinely used for the diagnostics of multiple pathogens, including *D. immitis*, *A. vasorum*, tick-borne pathogens and *Giardia* [65], although not easily accessible in low-income countries [66].

Computer-based algorithms to identify parasites in microscopy-based faecal examinations have recently been developed and demonstrate a similar qualitative performance to the parasitologists' eye with conventional faecal flotation techniques [67,68]. Smartphone apps and electronic maps with data on parasite distribution are used not only by research groups but also by end users [69,70]. Risk maps developed by scientific councils or private diagnostic companies serve as a general representation of the parasite activity and risk for given areas. Data are designed to show the proportion of pets positive for a given infection, allowing to better estimate the exposure to a parasite threat and design better CAP prevention advices during travel. Similarly, citizen science apps and project such as Tekenscanner in The Netherlands [70], or ZanzaMapp in Italy allow to record, identify ticks and test them for pathogens [71] and to assess citizens' perception of mosquito abundance and nuisance to feed spatial analyses for monitoring and control by local administrations [71]. Such tools will increasingly support veterinarians for faster and more reliable diagnosis, and scientists to investigate new emerging parasites and epidemiological trends.

### 6.3 Best practices and veterinary advice

Best practice regarding the prevention and treatment of parasites in pets are promoted through guidelines released by specific scientific councils which operate in different regions of the world, such as the European Scientific Counsel Companion Animal Parasites (ESCCAP) [72], the Companion Animal Parasite Council (CAPC) in the USA [73], and the Tropical Council for Companion Animal Parasites (TroCCAP) [74]. The guidelines, derived from solid investigations and applied field research, are continually revised based on the evolution of parasite epidemiology and applicable legislation. All together, these procedures support veterinary professionals and animal owners, with a relevant/proven impact on the control of canine and feline parasites [75].

**Fig. 2:**
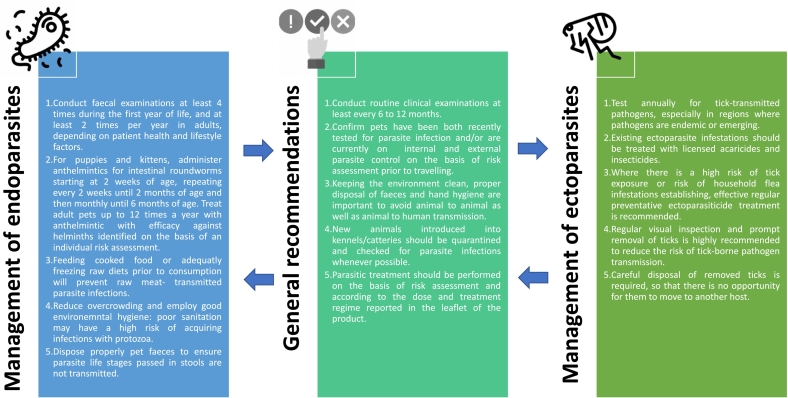
Best veterinary practice regarding prevention and treatment in companion animals. Information is based on Guidelines developed by ESCCAP, with a focus on the general recommendations and on management of endo- and ectoparasites.

Considering that both management plans and treatment regimens depend on local legislation, availability of registered medicinal products at national level and the epidemiological situation of given parasitic infections, lifelong control of common parasites of dogs and cats is generally encouraged by parasite councils as only a few infections are strictly age-related [[72], [73], [74]]. The principle to administer an effective parasiticide applies to all pets, being refined based on individual assessments and calibrated risk-based approach, i.e. the lifestyle and behaviour as well as the geographical location of the animal [[72], [73], [74]]. For instance, owned dogs with no outdoors contact with other animals, parks, sandpits, playgrounds, or gastropods are allocated to a low-risk category for round- and tapeworms, thus reducing the diagnostic and treatment practice to a periodicity of 1–2 times a year [72] (Fig. 2).

Despite the existence of these recommendations, surveys on the perception of these messages by owners often outline a non-compliance with the proposed indications [76,77]. An European study showed that only 2% of dogs and no cats were being dewormed 4 times a year, despite 93% of dogs and 54% of cats falling into the highest risk group requiring at least this frequency [76]. In addition, animal owners may not fully perceive the risk posed by some potentially life-threatening parasites (such as *D. immitis* or *A. vasorum* in dogs), which together with the zoonotic *Toxocara* sp. and *Echinococcus* sp. are considered the key pet parasites of major concern [76,77]. The owner choice of a parasiticide is mainly driven by the selling price of the product, spectrum of action and treatment regime. Preference for a spot on, tablet or collar also plays a role as the mode of application can affect the confidence and willingness of an owner to apply it [76,77]. The understanding of pet owners towards parasite risks may also vary based on sociodemographic factors, as demonstrated in Australia where female respondents scored greater that males; or according to duration of animal ownership, which is generally positively linked to an improved appreciation about zoonoses [78]. Regardless of stats and figures, an increased frequency in visiting veterinary clinics is positively associated with likelihood of owners performing antiparasitic treatments, proper faecal disposal, and cooking meat before feeding to animals [78]. Engagement of pet owners with veterinary clinics is also beneficial in reinforcing the One Health role of scientific councils and veterinary professionals in the era of the information technology revolution.

## 7 Concluding remarks

Looking at CAPs through a One Health lens allowed to identify the existence of numerous unmet needs associated to their control. Current trends suggest that multimodal approaches for a responsible control of CAPs deserve dedicated attention. This should prompt end-users, industry, and governmental bodies to prioritise the discussion of action plans for integrated control tools across the veterinary, medical and environmental disciplines. In particular:

(1) Climate change and globalisation are altering the health and welfare conditions of pet animals and their owners. There is a need for improving surveillance, prediction and awareness systems supported by insights from basic research on how global changes are altering parasite epidemiology and transmission between pets and humans.
(2) There are hardly any quantitative indicators assessing the impact of CAP control to the benefit of human, animal and environmental health. We need more cost-benefit and benefit-risk assessments at the local and societal level.
(3) Recommendations regarding responsible pet ownership, including importance of hygienic practices, are key for mitigating (re-)emerging parasite infections. We need better knowledge of pet owner perceptions to develop adapted communications strategies.
(4) Future control approaches should adopt new possibilities in diagnostics and risk prediction to prepare for the changing challenges in pet parasite control.

## Funding

This study received financial support from HealthforAnimals.

## Declaration of competing interest

Alessio Giannelli is employed at Inovet (Belgium). All other authors declare that they have no competing interests.

## Data Availability

No data was used for the research described in the article.

## References

[1] Krouzecky C., Aden J., Hametner K., Klaps A., Kovacovsky Z., Stetina B.U. (2022). Fantastic beasts and why it is necessary to understand our relationship-animal companionship under challenging circumstances using the example of long-Covid. Animals (Basel).

[2] GFK (2016). Man’s Best Friend: Global Pet Ownership and Feeding Trends. https://www.gfk.com/insights/mans-best-friend-global-pet-ownership-and-feeding-trends.

[3] Health For Animals (2022). Global Trends in the Animal Health Sector. https://www.healthforanimals.org/reports/global-trends-in-the-animal-health-sector/.

[4] Selzer P.M., Epe C. (2021). Antiparasitics in animal health: quo Vadis?. Trends Parasitol..

[5] Animal Health Europe (2022). Key Figures. https://animalhealtheurope.eu/about-us/annual-reports/2022-2/key-figures-2022.

[6] Takashima G.K., Day M.J. (2014). Setting the one health agenda and the human-companion animal bond. Int. J. Environ. Res. Public Health.

[7] Overgaauw P.A.M., Vinke C.M., Hagen M.A.E.V., Lipman L.J.A. (2020). A one health perspective on the human-companion animal relationship with emphasis on zoonotic aspects. Int. J. Environ. Res. Public Health.

[8] Taylor M.A., Coop R.L., Wall R.L., Taylor M.A., Coop R.L., Wall R.L. (2015). Veterinary Parasitology.

[9] Deplazes P., Eckert J., Mathis A., von Samson-Himmelstjerna G., Zahner H. (2016).

[10] Bowman D., Fogarty E., Barr S.C. (2002).

[11] Otranto D. (2018). Arthropod-borne pathogens of dogs and cats: from pathways and times of transmission to disease control. Vet. Parasitol..

[12] Baneth G., Solano-Gallego L. (2022). Leishmaniasis. Vet. Clin. North Am. Small Anim. Pract..

[13] Iannino F., Sulli N., Maitino A., Pascucci I., Pampiglione G., Salucci S. (2017). Fleas of dog and cat: species, biology and flea-borne diseases. Vet. Ital..

[14] Huertas-López A., Álvarez-García G., Sánchez-Sánchez R., Cantos-Barreda A., Ibáñez-López F.J., Martínez-Subiela S., Cerón J.J., Martínez-Carrasco C. (2023). A systematic review and meta-analysis of the serological diagnosis of *toxoplasma gondii* infection highlight the lack of a one health integrative research. Res. Vet. Sci..

[15] Carlin E.P., Tyungu D.L. (2020). *Toxocara*: protecting pets and improving the lives of people. Adv. Parasitol..

[16] Carmena D., Cardona G.A. (2020). Echinococcosis in wild carnivorous species: epidemiology, genotypic diversity, and implications for veterinary public health. Vet. Parasitol..

[17] Couret J., Schofield S., Narasimhan S. (2022). The environment, the tick, and the pathogen - it is an ensemble. Front. Cell. Infect. Microbiol..

[18] Colella V., Bradbury R., Traub R. (2021). Ancylostoma ceylanicum. Trends Parasitol..

[19] Dantas-Torres F., Ketzis J., Pérez Tort G., Mihalca A.D., Baneth G., Otranto D., Watanabe M., Linh B.K., Inpankaew T., Borrás P., Arumugam S., Penzhorn B.L., Ybañez A.P., Irwin P., Traub R.J. (2023). Heartworm adulticide treatment: a tropical perspective. Parasit. Vectors.

[20] Kachani M., Heath D. (2014). Dog population management for the control of human echinococcosis. Acta Trop..

[21] Thompson R.C. (2013). Parasite zoonoses and wildlife: one health, spillover and human activity. Int. J. Parasitol..

[22] Deplazes P., van Knapen F., Schweiger A., Overgaauw P.A. (2011). Role of pet dogs and cats in the transmission of helminthic zoonoses in Europe, with a focus on echinococcosis and toxocarosis. Vet. Parasitol..

[23] Tsao J.I., Hamer S.A., Han S., Sidge J.L., Hickling G.J. (2021). The contribution of wildlife hosts to the rise of ticks and tick-borne diseases in North America. J. Med. Entomol..

[24] Denk D., De Neck S., Khaliq S., Stidworthy M.F. (2022). Toxoplasmosis in zoo animals: a retrospective pathology review of 126 cases. Animals (Basel)..

[25] Dubey J.P., Murata F.H.A., Cerqueira-Cézar C.K., Kwok O.C.H., Grigg M.E. (2020). Recent epidemiologic and clinical importance of *toxoplasma gondii* infections in marine mammals: 2009-2020. Vet. Parasitol..

[26] Di Bari C., Venkateswaran N., Fastl C., Gabriël S., Grace D., Havelaar A.H., Huntington B., Patterson G.T., Rushton J., Speybroeck N., Torgerson P., Pigott D.M., Devleesschauwer B. (2023). The global burden of neglected zoonotic diseases: current state of evidence. One Health..

[27] Baneth G., Thamsborg S.M., Otranto D., Guillot J., Blaga R., Deplazes P., Solano-Gallego L. (2016). Major parasitic Zoonoses associated with dogs and cats in Europe. J. Comp. Pathol..

[28] Maia C., Conceição C., Pereira A., Rocha R., Ortuño M., Muñoz C., Jumakanova Z., Pérez-Cutillas P., Özbel Y., Töz S., Baneth G., Monge-Maillo B., Gasimov E., Van der Stede Y., Torres G., Gossner C.M., Berriatua E. (2023). The estimated distribution of autochthonous leishmaniasis by *Leishmania infantum* in Europe in 2005-2020. PLoS Negl. Trop. Dis..

[29] Dantas-Torres F., Miró G., Baneth G., Bourdeau P., Breitschwerdt E., Capelli G., Cardoso L., Day M.J., Dobler G., Ferrer L., Irwin P., Jongejan F., Kempf V.A.J., Kohn B., Lappin M., Little S., Madder M., Maggi R., Maia C., Marcondes M., Naucke T., Oliva G., Pennisi M.G., Penzhorn B.L., Peregrine A., Pfeffer M., Roura X., Sainz A., Shin S., Solano-Gallego L., Straubinger R.K., Tasker S., Traub R., Wright I., Bowman D.D., Gradoni L., Otranto D. (2019). Canine Leishmaniasis control in the context of one health. Emerg. Infect. Dis..

[30] Martín-Sánchez J., Rodríguez-Granger J., Morillas-Márquez F., Merino-Espinosa G., Sampedro A., Aliaga L., Corpas-López V., Tercedor-Sánchez J., Aneiros-Fernández J., Acedo-Sánchez C., Porcel-Rodríguez L., Díaz-Sáez V. (2020). Leishmaniasis due to *Leishmania infantum*: integration of human, animal and environmental data through a one health approach. Transbound. Emerg. Dis..

[31] Woolsey I.D., Miller A.L. (2021). *Echinococcus granulosus* sensu lato and *Echinococcus multilocularis*: a review. Res. Vet. Sci..

[32] Budke C.M., Casulli A., Kern P., Vuitton D.A. (2017). Cystic and alveolar echinococcosis: successes and continuing challenges. PLoS Negl. Trop. Dis..

[33] Romig T., Deplazes P., Jenkins D., Giraudoux P., Massolo A., Craig P.S., Wassermann M., Takahashi K., de la Rue M. (2017). Ecology and life cycle patterns of *Echinococcus* species. Adv. Parasitol..

[34] Abdullah S., Helps C., Tasker S., Newbury H., Wall R. (2019). Pathogens in fleas collected from cats and dogs: distribution and prevalence in the UK. Parasit. Vectors.

[35] Bitam I., Dittmar K., Parola P., Whiting M.F., Raoult D. (2010). Fleas and flea-borne diseases. Int. J. Infect. Dis..

[36] Morgan E.R. (2016). Risks from emerging parasitic zoonoses in companion animals. Comp. Animal..

[37] Charlier J., Barkema H.W., Becher P., De Benedictis P., Hansson I., Hennig-Pauka I., La Ragione R., Larsen L.E., Madoroba E., Maes D., Marín C.M., Mutinelli F., Nisbet A.J., Podgórska K., Vercruysse J., Vitale F., Williams D.J.L., Zadoks R.N. (2022). Disease control tools to secure animal and public health in a densely populated world. Lancet Planet Health..

[38] Short E.E., Caminade C., Thomas B.N. (2017). Climate change contribution to the emergence or re-emergence of parasitic diseases. Infect. Dis. (Auckl)..

[39] Pozio E. (2020). How globalization and climate change could affect foodborne parasites. Exp. Parasitol..

[40] Mordecai E.A., Caldwell J.M., Grossman M.K., Lippi C.A., Johnson L.R., Rohr M. Neira M.J.R., Ryan S.J., Savage V., Shocket M.S., Sippy R., Ibarra A.M. Stewart, Thomas M.B., Villena O. (2019). Thermal biology of mosquito-borne disease. Ecol. Lett..

[41] Semenza J.C., Rocklöv J., Ebi K.L. (2022). Climate change and cascading risks from infectious disease. Infect. Dis. Ther..

[42] Protopopova A., Ly L.H., Eagan B.H., Brown K.M. (2021). Climate change and companion animals: identifying links and opportunities for mitigation and adaptation strategies. Integr. Comp. Biol..

[43] European Centre for Disease Prevention and Control, Mosquito Maps. 2023 https://www.ecdc.europa.eu/en/disease-vectors/surveillance-and-disease-data/mosquito-maps (accessed 15 August 2022).

[44] Genchi C., Rinaldi L., Mortarino M., Genchi M., Cringoli G. (2009). Climate and *Dirofilaria* infection in Europe. Vet. Parasitol..

[45] Anvari D., Narouei E., Daryani A., Sarvi S., Moosazadeh M., Ziaei Hezarjaribi H., Narouei M.R., Gholami S. (2020). The global status of *Dirofilaria immitis* in dogs: a systematic review and meta-analysis based on published articles. Res. Vet. Sci..

[46] Genchi C., Kramer L.H. (2020). The prevalence of *Dirofilaria immitis* and *D. Repens* in the Old World. Vet. Parasitol..

[47] Oliveira S.V., Romero-Alvarez D., Martins T.F., Santos J.P.D., Labruna M.B., Gazeta G.S., Escobar L.E., Gurgel-Gonçalves R. (2017). *Amblyomma* ticks and future climate: range contraction due to climate warming. Acta Trop..

[48] Ryan S.J., McNally A., Johnson L.R., Mordecai E.A., Ben-Horin T., Paaijmans K., Lafferty K.D. (2015). Mapping physiological suitability limits for malaria in Africa under climate change. Vect. Borne Zoonot. Dis..

[49] Loeb J. (2021). New pet travel rules ‘a good thing’ for cats. Vet. Rec..

[50] Wright I., Jongejan F., Marcondes M., Peregrine A., Baneth G., Bourdeau P., Bowman D.D., Breitschwerdt E.B., Capelli G., Cardoso L., Dantas-Torres F., Day M.J., Dobler G., Ferrer L., Gradoni L., Irwin P., Kempf V.A.J., Kohn B., Krämer F., Lappin M., Madder M., Maggi R.G., Maia C., Miró G., Naucke T., Oliva G., Otranto D., Pennisi M.G., Penzhorn B.L., Pfeffer M., Roura X., Sainz A., Shin S., Solano-Gallego L., Straubinger R.K., Tasker S., Traub R., Little S. (2020). Parasites and vector-borne diseases disseminated by rehomed dogs. Parasit. Vectors.

[51] Buczek A., Buczek W. (2020). Importation of ticks on companion animals and the risk of spread of tick-borne diseases to non-endemic regions in Europe. Animals (Basel)..

[52] Gu D., Andreev K., Dupre M.E. (2021). Major trends in population growth around the world. China CDC Wkly..

[53] Dantas-Torres F. (2015). Climate change, biodiversity, ticks and tick-borne diseases: the butterfly effect. Int. J. Parasitol. Parasit. Wildl..

[54] Hotez P. (2022). Communicating science and protecting scientists in a time of political instability. Trends Mol. Med..

[55] Drake J., Parrish R.S. (2019). Dog importation and changes in heartworm prevalence in Colorado 2013-2017. Parasit. Vectors.

[56] Mettler M., Grimm F., Naucke T.J., Maasjost C., Deplazes P. (2005). Canine leishmaniosis in Central Europe: retrospective survey and serological study of imported and travelling dogs. Berl. Munch. Tierarztl. Wochenschr..

[57] Maggi R.G., Halls V., Krämer F., Lappin M., Pennisi M.G., Peregrine A.S., Roura X., Schunack B., Scorza V., Tasker S., Baneth G., Bourdeau P., Bowman D.D., Breitschwerdt E.B., Capelli G., Cardoso L., Dantas-Torres F., Dobler G., Ferrer L., Gradoni L., Irwin P., Jongejan F., Kempf V.A.J., Kohn B., Little S., Madder M., Maia C., Marcondes M., Miró G., Naucke T., Oliva G., Otranto D., Penzhorn B.L., Pfeffer M., Sainz A., Shin S., Solano-Gallego L., Straubinger R.K., Traub R., Wright I. (2022). Vector-borne and other pathogens of potential relevance disseminated by relocated cats. Parasit. Vectors.

[58] Noack S., Harrington J., Carithers D.S., Kaminsky R., Selzer P.M. (2021). Heartworm disease - overview, intervention, and industry perspective. Int. J. Parasitol. Drugs Drug Resist..

[59] von Samson-Himmelstjerna G., Thompson R.A., Krücken J., Grant W., Bowman D.D., Schnyder M., Deplazes P. (2021). Spread of anthelmintic resistance in intestinal helminths of dogs and cats is currently less pronounced than in ruminants and horses - yet it is of major concern. Int. J. Parasitol. Drugs Drug Resist..

[60] Charlier J., Bartley D.J., Sotiraki S., Martinez-Valladares M., Claerebout E., von Samson-Himmelstjerna G., Thamsborg S.M., Hoste H., Morgan E.R., Rinaldi L. (2022). Anthelmintic resistance in ruminants: challenges and solutions. Adv. Parasitol..

[61] Prichard R.K. (2021). Macrocyclic lactone resistance in *Dirofilaria immitis*: risks for prevention of heartworm disease. Int. J. Parasitol..

[62] Marsh A.E., Lakritz J. (2023). Reflecting on the past and fast forwarding to present day anthelmintic resistant *Ancylostoma caninum*-a critical issue we neglected to forecast. Int. J. Parasitol. Drugs Drug Resist..

[63] Venkatesan A., Jimenez Castro P.D., Morosetti A., Horvath H., Chen R., Redman E., Dunn K., Collins J.B., Fraser J.S., Andersen E.C., Kaplan R.M., Gilleard J.S. (2023). Molecular evidence of widespread benzimidazole drug resistance in *Ancylostoma caninum* from domestic dogs throughout the USA and discovery of a novel β-tubulin benzimidazole resistance mutation. PLoS Pathog..

[64] Khurana S., Singh S., Mewara A. (2021). Diagnostic techniques for soil-transmitted helminths - recent advances. Res. Rep. Trop. Med..

[65] Momčilović S., Cantacessi C., Arsić-Arsenijević V., Otranto D., Tasić-Otašević S. (2019). Rapid diagnosis of parasitic diseases: current scenario and future needs. Clin. Microbiol. Infect..

[66] Lorusso V. (2021). Parasitology and one health-perspectives on Africa and beyond. Pathogens..

[67] Nagamori Y., Sedlak R.H., DeRosa A., Pullins A., Cree T., Loenser M., Larson B.S., Smith R.B., Penn C., Goldstein R. (2021). Further evaluation and validation of the VETSCAN IMAGYST: in-clinic feline and canine fecal parasite detection system integrated with a deep learning algorithm. Parasit. Vectors.

[68] Rinaldi L., Krücken J., Martinez-Valladares M., Pepe P., Maurelli M.P., de Queiroz C., de Agüero V. Castilla Gómez, Wang T., Cringoli G., Charlier J., Gilleard J.S., von Samson-Himmelstjerna G. (2022). Advances in diagnosis of gastrointestinal nematodes in livestock and companion animals. Adv. Parasitol..

[69] Braks M., Schaffner F., Medlock J.M., Berriatua E., Balenghien T., Mihalca A.D., Hendrickx G., Marsboom C., Van Bortel W., Smallegange R.C., Sprong H., Gossner C.M., Czwienczek E., Dhollander S., Briët O., Wint W. (2022). VectorNet: putting vectors on the map. Front. Public Health.

[70] Kooyman F.N.J., Zweerus H., Nijsse E.R., Jongejan F., Wagenaar J.A., Broens E.M. (2022). Monitoring of ticks and their pathogens from companion animals obtained by the “tekenscanner” application in the Netherlands. Parasitol. Res..

[71] Caputo B., Manica M., Filipponi F., Blangiardo M., Cobre P., Delucchi L., De Marco C.M., Iesu L., Morano P., Petrella V., Salvemini M., Bianchi C., Torre A. Della (2020). ZanzaMapp: a scalable citizen science tool to monitor perception of mosquito abundance and nuisance in Italy and beyond. Int. J. Environ. Res. Public Health.

[72] European Scientific Counsel Companion Animal Parasites (ESCCAP) (2024). Guidelines. https://www.esccap.org/guidelines/.

[73] Companion Animal Parasite Council (CAPC) (2024). Guidelines. https://capcvet.org/guidelines/.

[74] Dantas-Torres F., Ketzis J., Mihalca A.D., Baneth G., Otranto D., Tort G.P., Watanabe M., Linh B.K., Inpankaew T., Jimenez Castro P.D., Borrás P., Arumugam S., Penzhorn B.L., Ybañez A.P., Irwin P., Traub R.J. (2020). TroCCAP recommendations for the diagnosis, prevention and treatment of parasitic infections in dogs and cats in the tropics. Vet. Parasitol..

[75] Vrhovec M.G., Alnassan A.A., Pantchev N., Bauer C. (2022). Is there any change in the prevalence of intestinal or cardiopulmonary parasite infections in companion animals (dogs and cats) in Germany between 2004-2006 and 2015-2017? An assessment of the impact of the first ESCCAP guidelines. Vet. Parasitol..

[76] McNamara J., Drake J., Wiseman S., Wright I. (2018). Survey of European pet owners quantifying endoparasitic infection risk and implications for deworming recommendations. Parasit. Vectors.

[77] Pennelegion C., Drake J., Wiseman S., Wright I. (2020). Survey of UK pet owners quantifying internal parasite infection risk and deworming recommendation implications. Parasit. Vectors.

[78] Bebrysz M., Wright A., Greaves M., Rathwell Deault D., Hopkins G., Gildea E., Aballéa S. (2021). How pet owners choose antiparasitic treatments for their dogs: a discrete choice experiment. Prev. Vet. Med..

[79] Moretti N.S., Mortara R.A., Schenkman S. (2020). Trypanosoma cruzi. Trends Parasitol..

[80] Dixon B.R. (2021). *Giardia duodenalis* in humans and animals - transmission and disease. Res. Vet. Sci..

[81] Dubey J.P. (2019).

[82] Campero L.M., Basso W., Moré G., Fiorani F., Hecker Y.P., Echaide I., Cantón G.J., Cirone K.M., Campero C.M., Venturini M.C., Moore D.P. (2023). Neosporosis in Argentina: past, present and future perspectives. Vet. Parasit. Reg. Stud. Report..

[83] Chai J.Y., Jung B.K. (2022). General overview of the current status of human foodborne trematodiasis. Parasitology..

[84] Blair D. (2022). Lung flukes of the genus *Paragonimus*: ancient and re-emerging pathogens. Parasitology..

[85] Capelli G., Genchi C., Baneth B., Bourdeau P., Brianti E., Cardoso L., Danesi P., Fuehrer H.P., Giannelli A., Ionică A.M., Maia C., Modrý D., Montarsi F., Krücken J., Papadopoulos E., Petrić D., Pfeffer M., Savić S., Otranto D., Poppert S., Silaghi C. (2018). Recent advances on *Dirofilaria repens* in dogs and humans in Europe. Parasit. Vectors.

[86] Cambra-Pellejà M., Gandasegui J., Balaña-Fouce R., Muñoz J., Martínez-Valladares M. (2020). Zoonotic implications of *Onchocerca* species on human health. Pathogens..

[87] Otranto D., Mendoza-Roldan J.A., Dantas-Torres F. (2021). Thelazia callipaeda. Trends Parasitol..

[88] Rousseau J., Castro A., Novo T., Maia C. (2022). *Dipylidium caninum* in the twenty-first century: epidemiological studies and reported cases in companion animals and humans. Parasit. Vectors.

[89] Morelli S., Diakou A., Colombo M., Di Cesare A., Barlaam A., Dimzas D., Traversa D. (2021). Cat respiratory nematodes: current knowledge, novel data and warranted studies on clinical features, treatment and control. Pathogens..

[90] Schnyder M., Bilbrough G., Hafner C., Schaper R. (2017). *Angiostrongylus vasorum*, “the French heartworm”: a serological survey in dogs from France introduced by a brief historical review. Parasitol. Res..

[91] Mendoza Roldan J.A., Otranto D. (2023). Zoonotic parasites associated with predation by dogs and cats. Parasit. Vectors.

[92] Skotarczak B. (2018). The role of companion animals in the environmental circulation of tick-borne bacterial pathogens. Ann. Agric. Environ. Med..

[93] Baneth G. (2014). Tick-borne infections of animals and humans: a common ground. Int. J. Parasitol..

[94] Heylen D., Day M., Schunack B., Fourie J., Labuschange M., Johnson S., Githigia S.M., Akande F.A., Nzalawahe J.S., Tayebwa D.S., Aschenborn O., Marcondes M., Madder M. (2021). A community approach of pathogens and their arthropod vectors (ticks and fleas) in dogs of African sub-Sahara. Parasit. Vectors.

[95] Madder M., Day M., Schunack B., Fourie J., Labuschange M., van der Westhuizen W., Johnson S., Githigia S.M., Akande F.A., Nzalawahe J.S., Tayebwa D.S., Aschenborn O., Marcondes M., Heylen D. (2022). A community approach for pathogens and their arthropod vectors (ticks and fleas) in cats of sub-Saharan Africa. Parasit. Vectors.

[96] Maggi R.G., Krämer F. (2019). A review on the occurrence of companion vector-borne diseases in pet animals in Latin America. Parasit. Vectors.

[97] Irwin P.J., Jefferies R. (2004). Arthropod-transmitted diseases of companion animals in Southeast Asia. Trends Parasitol..

[98] Benelli G., Caselli A., Di Giuseppe G., Canale A. (2018). Control of biting lice, Mallophaga - a review. Acta Trop..

[99] Moroni B., Rossi L., Bernigaud C., Guillot J. (2022). Zoonotic episodes of Scabies: a global overview. Pathogens..

[100] Deplazes P., Hegglin D., Gloor S., Romig T. (2004). Wilderness in the city: the urbanization of *Echinococcus multilocularis*. Trends Parasitol.

